# Supplementary material for: Using firm-level supply chain networks to measure the speed of the energy transition
Source: Nat Commun. 2026 Feb 9;17:2529. doi: 10.1038/s41467-026-69358-4 (PMC12996292; doi:10.1038/s41467-026-69358-4)
Supplement: Supplementary file 1 — Supplementary Information [file 41467_2026_69358_MOESM1_ESM.pdf]

Supplementary Information

Using firm-level supply chain networks to measure the speed of the energy transition

Johannes Stangl[1], András Borsos[1,2,3], Stefan Thurner[1,4,5,6*]

[1]Complexity Science Hub, Metternichgasse 8, 1030 Vienna, Austria.
[2]National Bank of Hungary, Szabadság tér 9, 1054 Budapest, Hungary.
[3]Institute for New Economic Thinking, Manor Road Building, Manor Road, Oxford, OX1 3UQ, United Kingdom.
[4]Supply Chain Intelligence Institute Austria, Metternichgasse 8, 1030 Vienna, Austria.
[5]Medical University of Vienna, Spitalgasse 23, 1090 Vienna, Austria.
[6]Santa Fe Institute, Santa Fe, 1399 Hyde Park Rd, NM 75791, United States of America.

*Corresponding author(s). E-mail(s): stefan.thurner@meduniwien.ac.at

Contents

Supplementary Methods **3**

Supplementary Methods 1: Industry classification and sample composition 3

Supplementary Methods 2: Electricity mix of Hungary and forecasts until 2050 4

Supplementary Methods 3: Electricity, gas and fuel price evolution in Hungary 6

Supplementary Methods 4: Comparison of OLS and robust estimation 9

Supplementary Discussion **10**

Firm sample description 10

Comparison of firm sample to sectoral energy consumption data 12

Uncertainty analysis of the energy scenarios 19

Limitations in establishing causality between firm characteristics and transition behavior 21

Estimation of the effect of solar PV self-consumption 21

Influence of suppliers and customers on transitioning behaviour 25

Supplementary Methods

Supplementary Methods 1: Industry classification and sample composition

Supplementary Table 1 contains the full descriptions of the NACE 1-digit categories accompanying Fig. 2a in the main text. Supplementary Table 2 provides the number of firms (sample size n) contributing to each sectoral box plot in Fig. 2b of the main text.

Supplementary Table 1: NACE 1-digit industry category codes and their corresponding descriptions accompanying Fig. 2a in the main text (NACE, Nomenclature of Economic Activities).

industry category code	description
A	Agriculture, forestry and fishing
B	Mining and quarrying
C	Manufacturing
D	Electricity, gas, steam and air conditioning supply
E	Water supply; sewerage, waste management and remediation activities
F	Construction
G	Wholesale and retail trade; repair of motor vehicles and motorcycles
H	Transportation and storage
I	Accommodation and food service activities
J	Information and communication
L	Real estate activities
M	Professional, scientific and technical activities
N	Administrative and support service activities
O	Public administration and defence; compulsory social security
P	Education
Q	Human health and social work activities
R	Arts, entertainment and recreation
S	Other service activities

Supplementary Table 2: Number of firms, n by NACE 2-digit industry sector contributing to the sectoral box plots in Fig. 2b of the main text (NACE, Nomenclature of Economic Activities).

NACE 2-digit sector	n	NACE 2-digit sector	n
A01	1378	G47	4234
A02	48	H49	338
A03	19	H50	6
B05	1	H51	2
B06	1	H52	188
B07	1	H53	14
B08	39	I55	651
B09	5	I56	1695
C10	872	J58	74
C11	238	J59	35
C12	3	J60	20
C13	95	J61	97
C14	128	J62	203
C15	45	J63	15
C16	204	L68	2870
C17	81	M69	457
C18	183	M70	252
C20	109	M71	500
C21	32	M72	89
C22	467	M73	73
C23	217	M74	109
C24	67	M75	79
C25	1092	N77	99
C26	131	N78	43
C27	134	N79	73
C28	381	N80	77
C29	121	N81	124
C30	30	N82	161
C31	168	O84	8
C32	142	P85	82
C33	176	Q86	79
E36	46	Q87	7
E37	15	Q88	15
E38	150	R90	82
E39	10	R91	19
F41	644	R92	8
F42	315	R93	180
F43	706	S94	77
G45	1136	S95	76
G46	2140	S96	250

Supplementary Methods 2: Electricity mix of Hungary and forecasts until 2050

We use data from the online platform Ember, which provides annual data on clean and fossil electricity generation in terawatt-hours, to calculate the low-carbon share of Hungary's annual electricity mix $u(t)$ [1]. To estimate the future low-carbon share, we perform a linear regression based on the 2020-2024 observation period, during which the low-carbon share increased by 10%. This scenario, which would enable Hungary to reach its self-proclaimed target of 90% low-carbon electricity generation by 2030, requires an annual increase of $u(t)$ by 3.2%. In this scenario, Hungary's electricity grid would be essentially decarbonized by the year 2033. Supplementary Figure 1 and Supplementary Table 3 provide an overview of the evolution and the forecast of the low-carbon share $u(t)$ based on the electricity generation data.

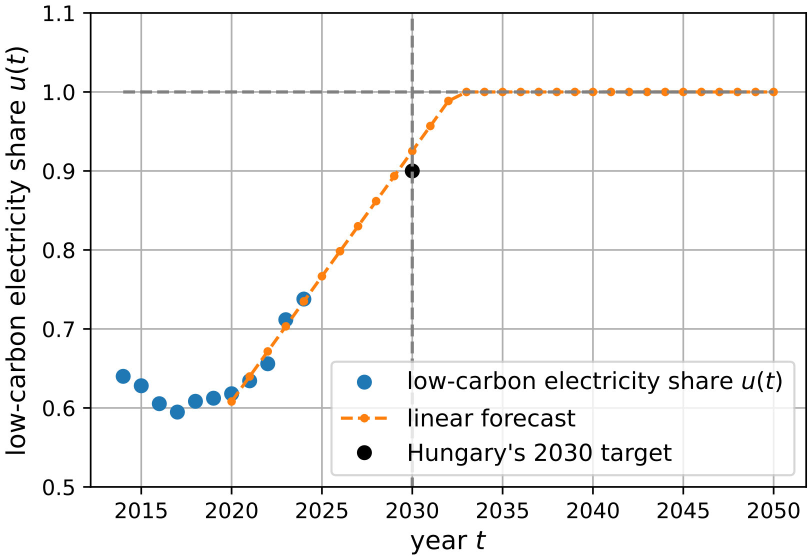

Supplementary Figure 1: Low-carbon share of the Hungarian electricity mix $u(t)$ for 2014–2024 and a forecast of its evolution until 2050, based on a linear regression fitted to data from 2020–2024. Hungary has set a target of 90% low-carbon electricity generation by 2030[2]. Exact values are provided in Supplementary Table 3.

Supplementary Table 3: Low-carbon share of the Hungarian electricity mix, $u(t)$, for 2014–2050. Observations for 2014–2024 are obtained from the online platform EMBER[1]. Values for 2025–2050 are forecasts based on a linear regression fitted to data from 2020–2024.

year	low-carbon share $u(t)$	type
2014	0.640	measured
2015	0.628	measured
2016	0.605	measured
2017	0.595	measured
2018	0.608	measured
2019	0.612	measured
2020	0.618	measured
2021	0.634	measured
2022	0.656	measured
2023	0.712	measured
2024	0.738	measured
2025	0.767	forecast
2026	0.798	forecast
2027	0.830	forecast
2028	0.862	forecast
2029	0.893	forecast
2030	0.925	forecast
2031	0.957	forecast
2032	0.989	forecast
2033	1.000	forecast
2034	1.000	forecast
2035	1.000	forecast
2040	1.000	forecast
2050	1.000	forecast

Supplementary Methods 3: Electricity, gas and fuel price evolution in Hungary

Electricity and gas prices for non-household consumers are sourced from EUROSTAT [3,4]. These data, based on the reports of energy providers, reflect price trends across various energy consumption classes. Electricity prices are categorized into seven ranges, while gas prices are divided into six. The prices, provided in Hungarian Forint (HUF), are reported semi-annually. Supplementary Figure 2 and Supplementary Figure 3 depict the evolution of the electricity and the gas price for non-household consumers in Hungary. Since the supply chain network data are aggregated on a semi-annual basis, we can directly use the semi-annual price data to convert monetary values into kilowatt-hours of energy consumed. For firms with annual reporting requirements, however, we apply the average of the semi-annual prices within the year. This procedure may still result in some under- or overestimation. Since the price data reflect only averages per energy consumption range, our estimates may be imprecise for firms whose actual prices differ due to their specific sourcing strategies, such as retail purchases, power purchase agreements, or spot market trading.

In order to estimate oil consumption, we assume that firms primarily consume oil in the form of fuels, using fuel price trends as a proxy to convert observed oil expenditures into energy units. Data from the National Detailed Energy Balance, provided by the Hungarian Energy and Public Utility Regulatory Authority, confirms that diesel and gasoline are by far the most significant forms of oil product consumption [5]. Supplementary Figure 4 illustrates the distribution of oil product consumption for 2023, derived from the National Detailed Energy Balance data. Diesel accounts for nearly half of oil product consumption, followed by gasoline at 20%. Naphtha represents 14% of consumption; however, according to the National Detailed Energy Balance, the chemical and petrochemical industries are the sole consumers of naphtha.

Data on fuel prices is obtained from the historical price trends in the Weekly Oil Bulletin provided by the EU [6]. To determine a unique price for oil products, we calculate a weighted average of gasoline and diesel prices in Hungary. The weights are based on the relative consumption of gasoline and diesel in Hungary, also derived from the Weekly Oil Bulletin data, implicitly assuming that firms consume these fuels in similar proportions. In general, diesel represents about 74% of fuel consumption, whereas gasoline represents about 26% during the observation period from 2020 to 2024. We arrive at a unified price for fuel consumption in Hungary that allows us to convert monetary inputs for oil products into kilowatt-hours consumed. Supplementary Figure 5 depicts the evolution of fuel prices for gasoline, diesel, and their weighted average in Hungary for each semester from 2018 to 2024. Fuel prices have increased significantly since 2021, reflecting the broader energy crisis in Europe.

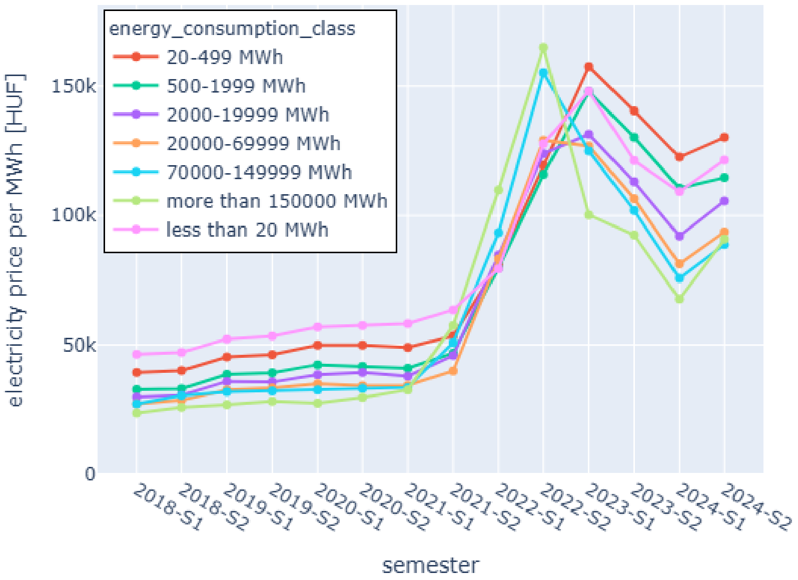

Supplementary Figure 2: Semi-annual electricity prices for non-household consumers in Hungary for 2018–2024, categorized by consumer size classes. Size classes are defined by annual electricity consumption in seven bands: less than 20 MWh, 20–499 MWh, 500–1,999 MWh, 2,000–19,999 MWh, 20,000–69,999 MWh, 70,000–149,999 MWh, and more than 150,000 MWh. Data are sourced from Eurostat [3].

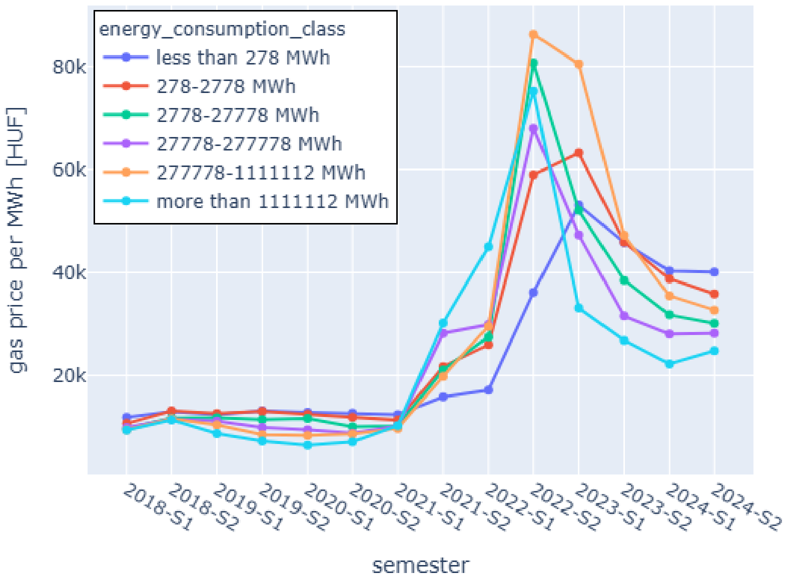

Supplementary Figure 3: Semi-annual gas prices for non-household consumers in Hungary for 2018–2024, categorized by consumer size classes. Size classes are defined by annual gas consumption in six bands: less than 278 MWh, 278–2,778 MWh, 2,778–27,778 MWh, 27,778–277,778 MWh, 277,778–1,111,112 MWh, and more than 1,111,112 MWh. Consumption bands are converted from gigajoules in the original dataset to megawatt-hours. Data are sourced from Eurostat [4].

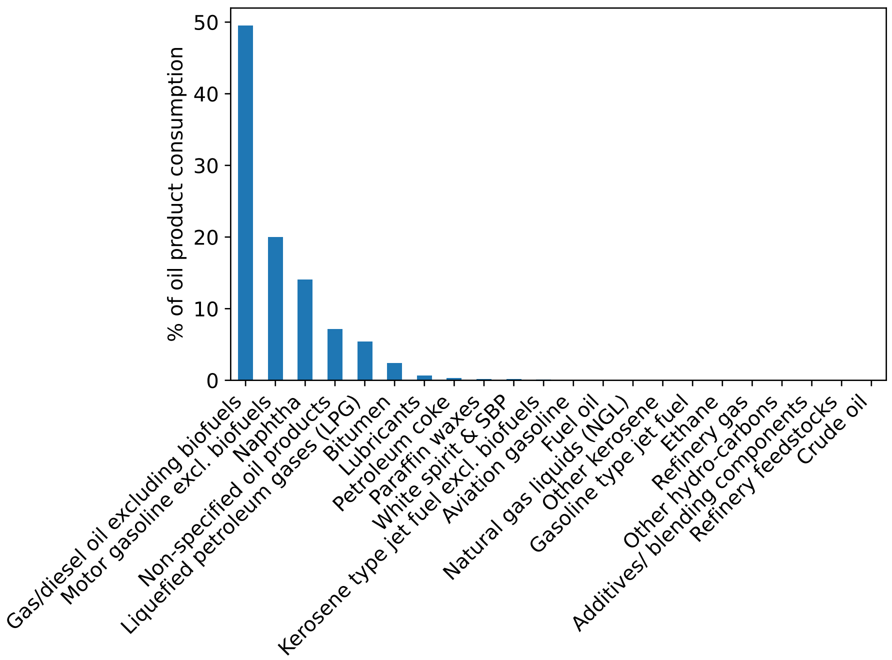

Supplementary Figure 4: Percentage shares of annual oil product consumption in 2023, derived from final consumption data in Hungary's National Detailed Energy Balance[5].

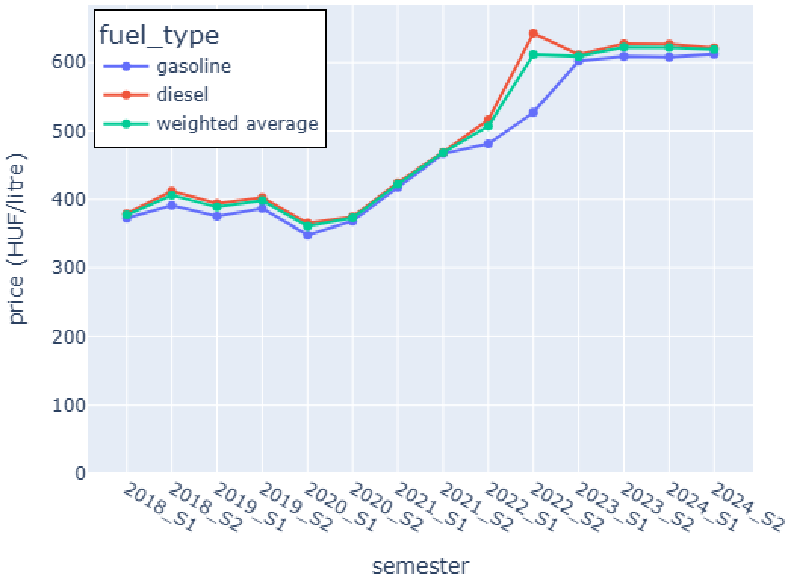

Supplementary Figure 5: Semi-annual fuel prices for non-household consumers in Hungary for 2018–2024. Prices for gasoline, diesel, and their weighted average are shown. Data are sourced from the Weekly Oil Bulletin of the European Commission[6].

Supplementary Methods 4: Comparison of OLS and robust estimation

Instead of an ordinary least squares (OLS) estimator, we employ a robust estimator based on the Huber norm[7], which down-weights outliers to obtain more reliable estimates of trends in the low-carbon share, $l_i(t)$. This approach is particularly valuable in the presence of crisis years within our observation period (2020–2024), such as the COVID-19 pandemic in 2020 or the energy crisis in 2022, and helps to mitigate potential firm-specific fluctuations. The details of the robust estimation procedure are provided in the Methods section of the main text. Supplementary Figure 6 compares the decarbonization trends, δ_i, and decarbonization rates, λ_i, estimated using OLS and robust regression. The robust estimator reduces sensitivity to outliers (e.g., Firm #2) while yielding results that are consistent with OLS when the data are well-behaved (e.g., Firm #1 and Firm #3).

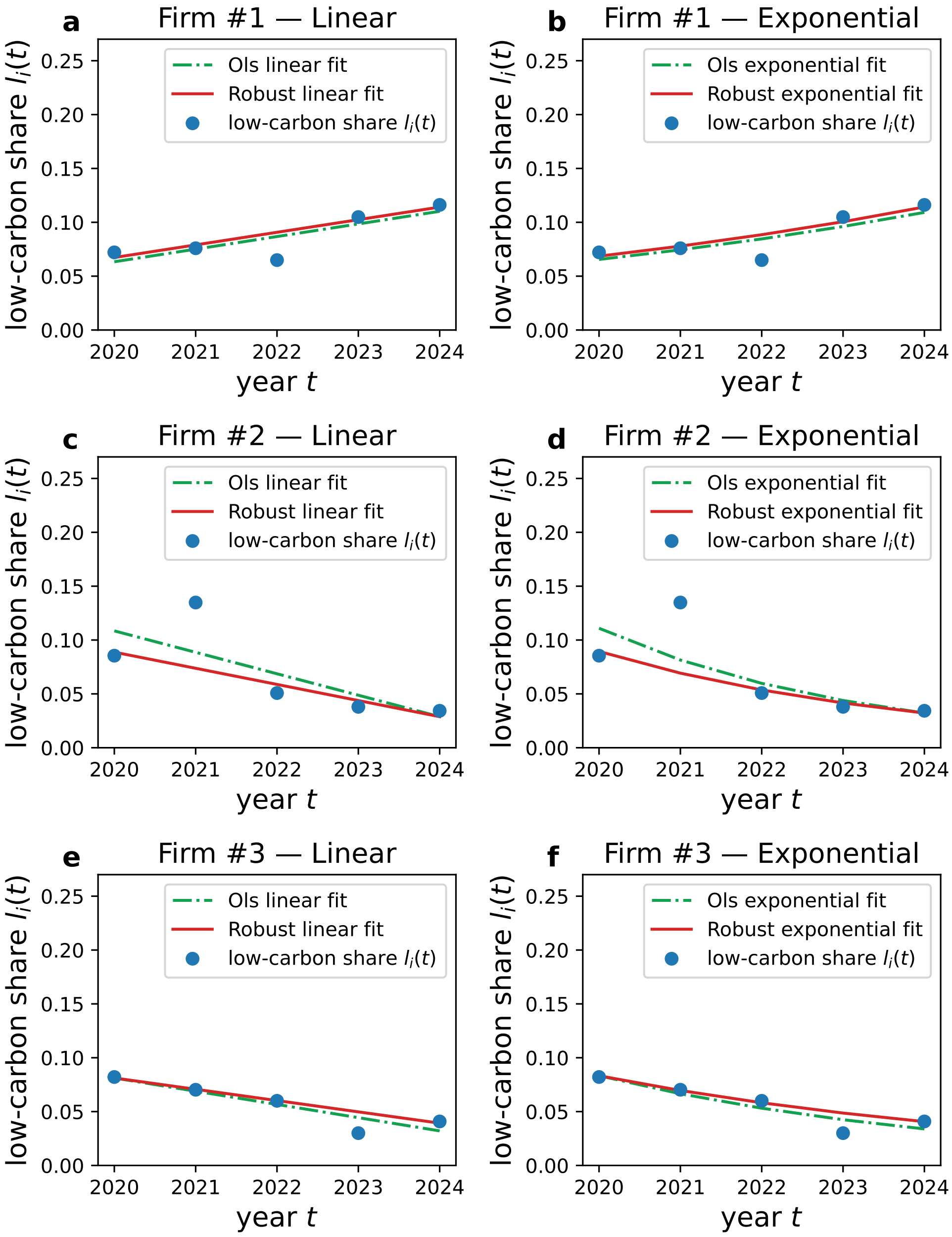

Supplementary Figure 6: Comparison of ordinary least squares (OLS) and robust regression fits (Huber loss) for linear (a, c, e) and exponential (b, d, f) models of the low-carbon share, $l_i(t)$, across three example firms. The robust estimator reduces sensitivity to outliers (e.g., Firm #2), while yielding trends consistent with OLS when the data are well behaved (e.g., Firms #1 and #3).

Supplementary Discussion

Firm sample description

We compare our constructed firm sample of 25,231 firms with the total firm dataset, which we treat as the 'ground truth,' to evaluate how well the sample represents the overall firm population in terms of energy inputs and revenue. Specifically, we compare the aggregated monetary inputs for gas, electricity, and oil, as well as the total revenue of firms in our sample, with the corresponding aggregated values in the total dataset. To ensure a fair comparison, we exclude energy-providing sectors, firms from the financial sector, firms covered by the emission trading system (ETS) and firms with no NACE category from the 'ground truth' dataset. However, we do not apply the time series consistency restrictions for energy inputs and revenue outlined in the Methods section on Firm sample construction. As a result, we exclude firms in the following NACE categories from the ground-truth sample: D35.1 (Electric power generation, transmission and distribution), including D35.1.1 (Production of electricity), D35.1.2 (Transmission of electricity), D35.1.3 (Distribution of electricity), and D35.1.4 (Trade of electricity); D35.2.1 (Manufacture of gas), D35.2.2 (Distribution of gaseous fuels through mains), and D35.2.3 (Trade of gas through mains); B06.10 (Extraction of crude petroleum); C19.20 (Manufacture of refined petroleum products); G47.30 (Retail sale of automotive fuel in specialised stores); G46.71 (Wholesale of solid, liquid and gaseous fuels and related products); K (Financial and insurance activities); and H52.21 (Service activities incidental to land transportation).

This results in a 'ground truth' dataset of 434,988 firms, which includes all firms regardless the continuity of energy inputs or revenue, except those in the excluded NACE categories or firms covered in the ETS. We then calculate the covered energy shares and revenue as fractions, defined as the ratio between the sums of the respective variables in our reconstructed firm sample and those in the 'ground truth' dataset. Supplementary Figure 7 illustrates the coverage, and Supplementary Table 4 provides further details. We achieve very good coverage for gas and electricity, with 75% coverage for gas inputs and approximately 70% for electricity inputs across all years. We also maintain good coverage for oil and revenue, with approximately 50% coverage for oil inputs across 2020-2024 and approximately 43% coverage for total revenue across 2020-2023. This means that our constructed firm sample captures a significant share of energy inputs and revenue from the total firm dataset, indicating that the sample consists of large and thus relevant firms.

To provide a more detailed description of our sample, we present the aggregated energy purchases (gas, oil, and electricity) of all firms in the sample from 2020 to 2024, along with their conversion into energy units (terawatt-hours, TWh) using energy prices, as shown in Supplementary Figure 8. As illustrated in Supplementary Figure 8a, energy purchases increased significantly in 2022 and 2023, coinciding with peak prices for gas, oil, and electricity. However, when these purchases are converted into energy units using energy prices, it becomes clear that total energy consumption across the different energy types remained relatively stable for the firms in the sample, as shown in Supplementary Figure 8b. Notably, electricity consumption appears to have declined since 2022.

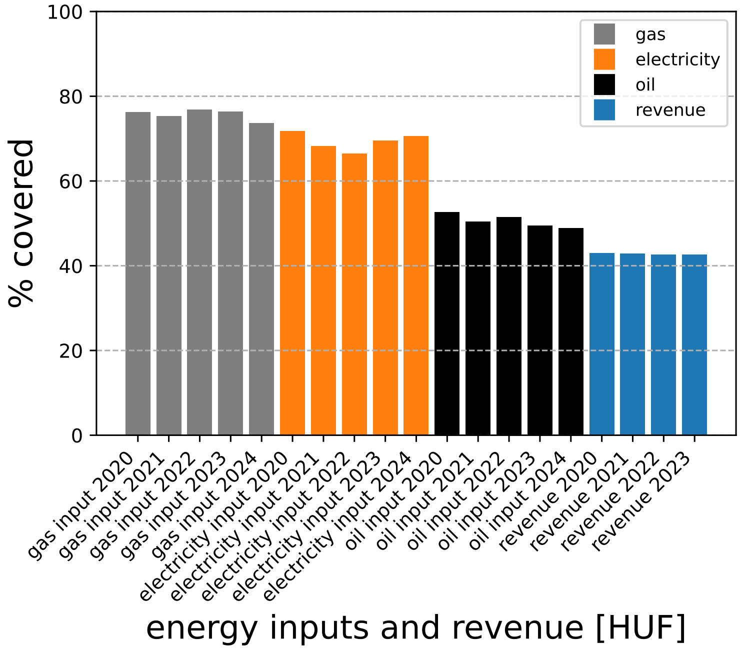

Supplementary Figure 7: Percentage coverage of the constructed firm sample relative to the full dataset. Bars represent the share of aggregated gas, electricity, and oil inputs (in Hungarian forints, HUF) for 2020–2024 and total revenue for 2020–2023 captured by the constructed sample. Exact values are provided in Supplementary Table 4.

Supplementary Table 4: Percentage coverage of the constructed firm sample relative to the full dataset, reported for aggregated gas, electricity, and oil inputs (in Hungarian forints, HUF) for 2020–2024 and total revenue for 2020–2023.

variable [HUF]	% covered
gas input 2020	76.22
gas input 2021	75.36
gas input 2022	76.84
gas input 2023	76.34
gas input 2024	73.62
electricity input 2020	71.74
electricity input 2021	68.22
electricity input 2022	66.46
electricity input 2023	69.56
electricity input 2024	70.66
oil input 2020	52.66
oil input 2021	50.38
oil input 2022	51.50
oil input 2023	49.45
oil input 2024	48.91
revenue 2020	42.97
revenue 2021	42.84
revenue 2022	42.70
revenue 2023	42.70

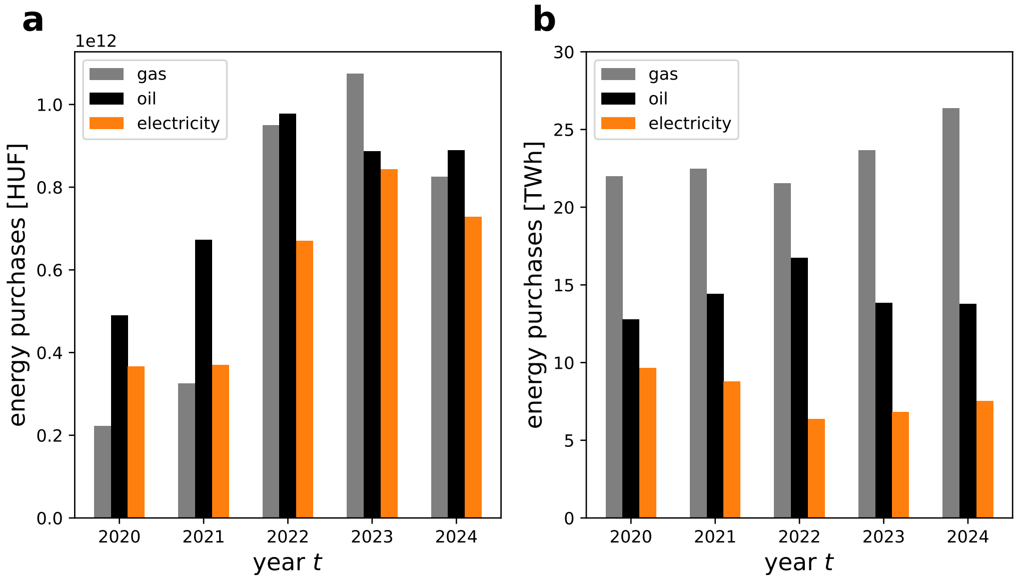

Supplementary Figure 8: Aggregated energy purchases by firms in the sample and their conversion to energy units for 2020–2024. (a) Aggregated monetary inputs for gas, electricity, and oil (in Hungarian forints, HUF) across all 25,231 firms in the sample. (b) Aggregated energy inputs for gas, electricity, and oil converted to terawatt-hours (TWh) across the same firms.

Comparison of firm sample to sectoral energy consumption data

We assess how well our firm sample represents sectoral energy consumption in Hungary by comparing it to official statistics. Specifically, we examine the total energy consumption (in TWh) of gas, electricity, and oil for the NACE 1-digit sectors 'A - Agriculture, forestry and fishing,' 'B - Mining and quarrying,' 'C - Manufacturing,' and 'F - Construction' using data from Hungary's energy balance[5]. Supplementary Figure 9 presents time series data for both the official statistics and our estimated energy consumption for the aggregated firm sample. Since our sample consists of only 25,231 firms, it is not expected to cover the total energy consumption of each sector. However, it should capture the relative consumption levels across sectors to some degree. As shown in Supplementary Figure 9, the sectoral distribution of gas, electricity, and oil consumption is well reflected in our estimates, with 'C - Manufacturing' consuming the most energy, followed by 'A - Agriculture, forestry and fishing,' and 'B - Mining and quarrying' consuming the least. However, our estimates indicate higher gas consumption and lower electricity consumption for 'C - Manufacturing' compared to official statistics. This suggests a possible underestimation of electricity consumption and an overestimation of gas consumption in our firm sample.

To further evaluate the accuracy of our estimates, we compare the share of electricity consumption in each NACE 1-digit sector using official energy balance data[5]. For each year between 2020 and 2024, we calculate the electricity share in the sectoral energy mix by dividing electricity consumption by the total consumption of electricity, natural gas, and oil. We then compare these shares to those in our firm sample. As shown in Supplementary Figure 10, our estimates generally capture the relative importance of electricity across sectors. 'C - Manufacturing' has the highest share of electricity in the energy mix, consistent with official statistics. However, our estimates show a lower overall electricity share, indicating a potential underestimation of electricity consumption. The discrepancy is particularly pronounced in 2022.

We additionally evaluate the same statistics for NACE 2-digit subsectors of the manufacturing sector, for which official data are available from Hungary's final energy use in industry for 2020–2023[8]. The sector labels in the official statistics were manually matched to NACE 2-digit codes. While the correspondence is largely one-to-one, some sectors represent aggregates of several NACE 2-digit codes. Supplementary Table 5 documents these correspondences. The relative importance of oil, gas, and electricity consumption across most manufacturing subsectors is preserved in our sample (Supplementary Figure 11). Gas consumption is reasonably well reflected,

whereas electricity appears underrepresented. As expected, the chemical sector is not well covered in oil use, since it also consumes oil in the form of naphtha and other products not captured in our analysis. Supplementary Figure 12 further compares electricity shares of manufacturing NACE 2-digit subsectors between the official statistics and our firm sample. While some of the relative importance across sectors is retained, the under-representation of electricity has a sizable impact on aggregate electricity shares. We suspect that firms in this sector may also procure electricity from the spot market, which cannot be captured by our method of assigning energy providers based on their NACE codes. Additionally, our analysis relies on a restricted sample of firms, and it remains inherently uncertain how much of the gap in electricity use relative to official statistics can be attributed to firms not captured in our data

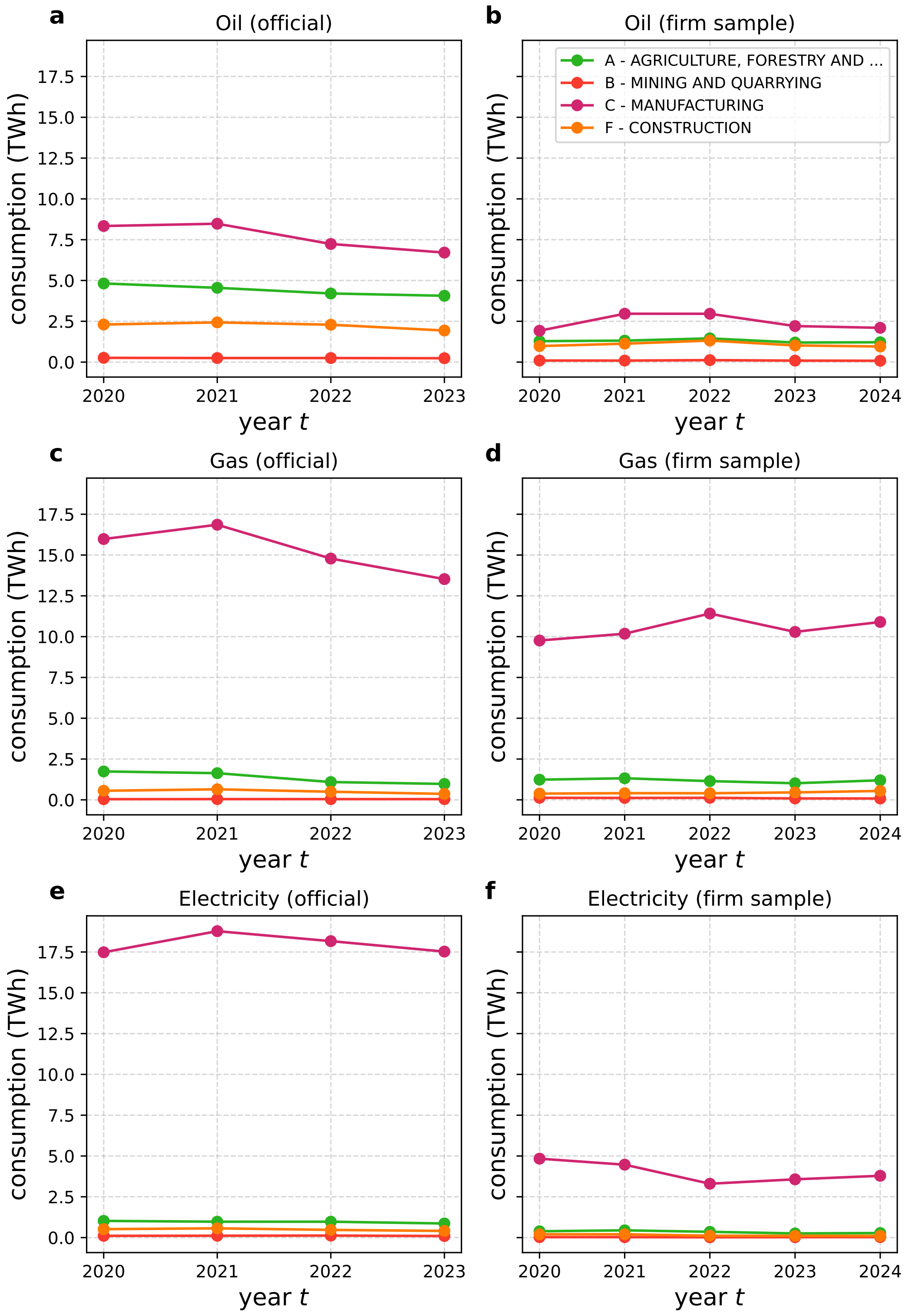

Supplementary Figure 9: Oil, gas, and electricity consumption by NACE 1-digit sectors, comparing Hungary's official energy balances[5] with estimates from the aggregated firm sample. The comparison covers sectors A (Agriculture, forestry and fishing), B (Mining and quarrying), C (Manufacturing), and F (Construction). Panels show electricity consumption from official statistics (a) and the firm sample (b), gas consumption from official statistics (c) and the firm sample (d), and oil consumption from official statistics (e) and the firm sample (f).

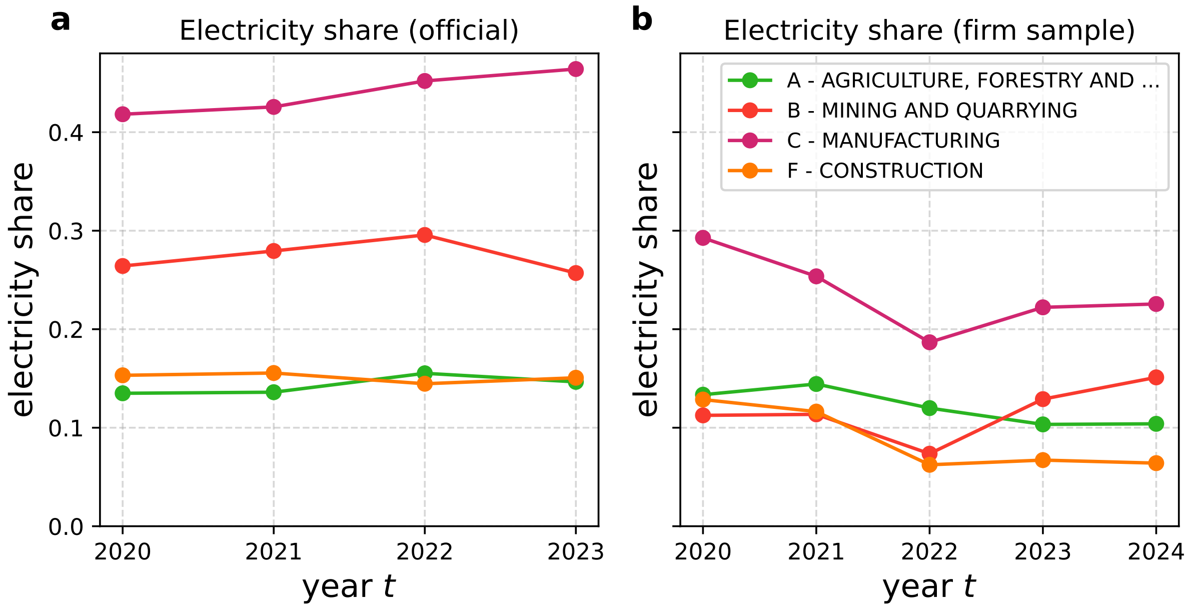

Supplementary Figure 10: Electricity shares of NACE 1-digit sectors, calculated from Hungary's energy balance[5] for 2020–2023 and compared with aggregated electricity shares from the firm sample. (a) Electricity shares for sectors A (Agriculture, forestry and fishing), B (Mining and quarrying), C (Manufacturing), and F (Construction) based on official statistics. (b) Corresponding aggregated electricity shares for the same sectors in the firm sample.

Supplementary Table 5: List of NACE 2-digit codes within sector C (Manufacturing) (NACE, Nomenclature of Economic Activities). Sector labels from Hungary's official statistics on final energy use in industry [8] were manually matched to NACE 2-digit codes. The correspondence is largely one-to-one; however, some sectors in the official statistics aggregate multiple NACE 2-digit codes and are therefore presented here in aggregated form.

NACE 2-digit code	Sector labels in official statistic
10, 11, 12	Food, Beverages and Tobacco
13, 14, 15	Textile and Leather
16	Wood and Wood Products (other than pulp and paper)
17, 18	Pulp, Paper and Printing
19, 20, 21	Chemical and petrochemical industries
22	Manufacture of rubber and plastic products
23	Non-Metallic Minerals: Glass, ceramic, cement and other building materials industries
24	Manufacture of basic metals
25	Manufacture of fabricated metal products, except machinery and equipment
26	Manufacture of computer, electronic and optical products
27	Manufacture of electrical equipment
28	Manufacture of machinery and equipment n.e.c.
29, 30	Transport Equipment
31	Manufacture of furniture
32	Other manufacturing

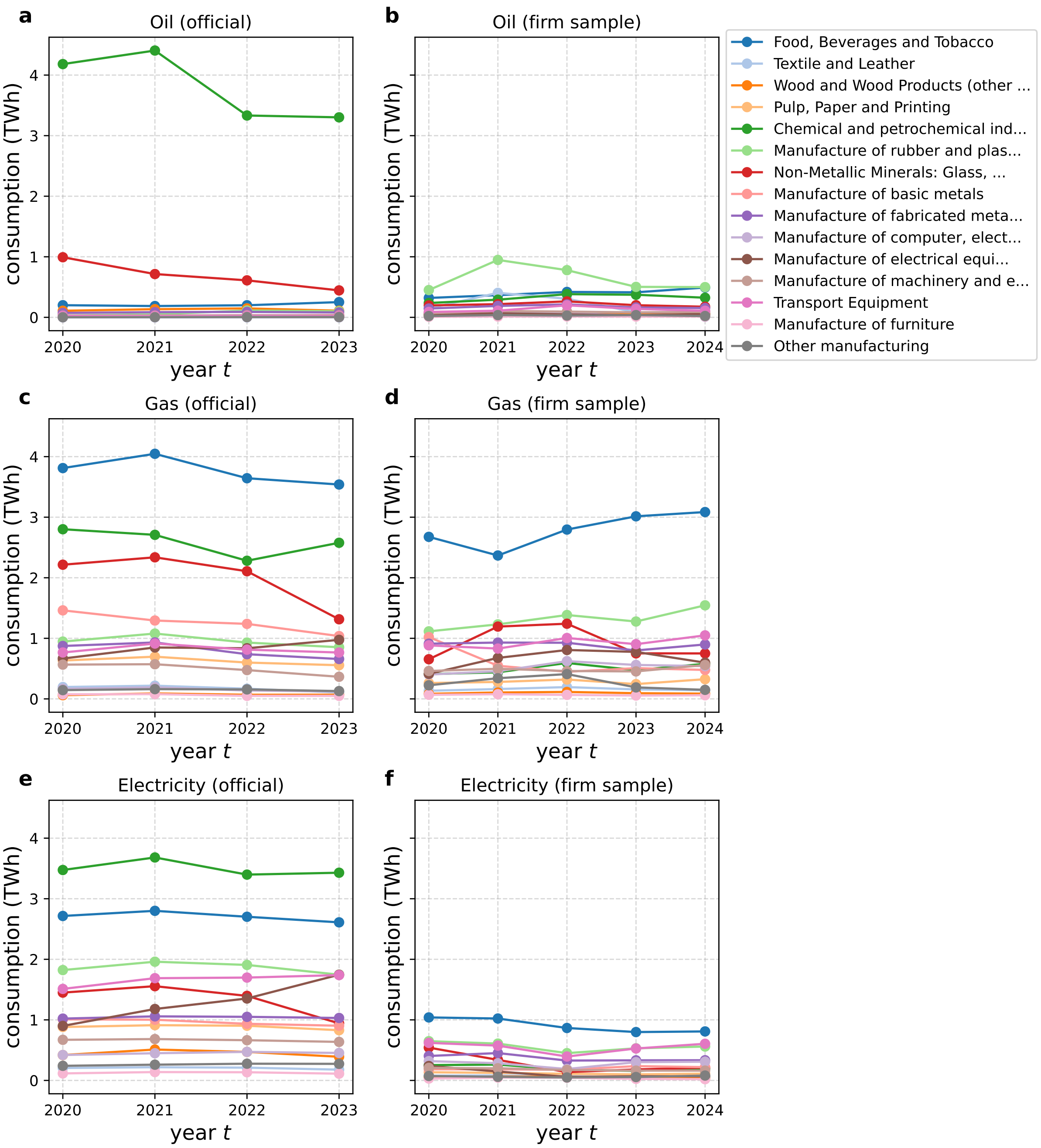

Supplementary Figure 11: Oil, gas, and electricity consumption by NACE 2-digit industries within sector C (Manufacturing) (NACE, Nomenclature of Economic Activities), comparing Hungary's official final energy use in industry for 2020–2023[8] (data for 2024 were not available at the time of analysis) with estimates from the aggregated firm sample for 2020–2024. Panels show electricity consumption from official statistics (a) and the firm sample (b), gas consumption from official statistics (c) and the firm sample (d), and oil consumption from official statistics (e) and the firm sample (f).

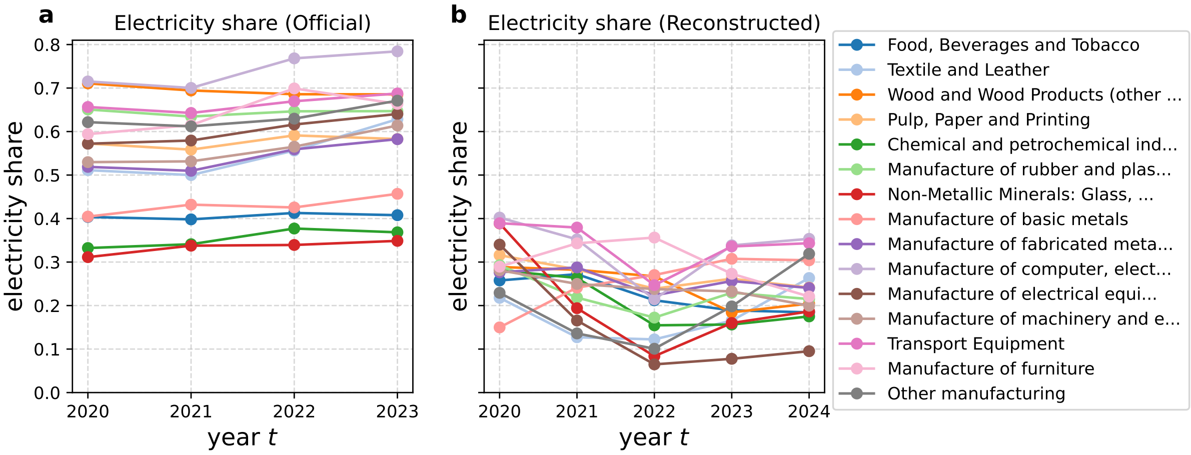

Supplementary Figure 12: Electricity shares by NACE 2-digit industries within sector C (Manufacturing) (NACE, Nomenclature of Economic Activities), comparing Hungary's official final energy use in industry for 2020–2023[8] with estimates from the aggregated firm sample for 2020–2024. (a) Electricity shares for manufacturing industries based on official statistics. (b) Corresponding aggregated electricity shares for the same industries in the firm sample.

Uncertainty analysis of the energy scenarios

To assess the uncertainty of the energy scenarios, we apply a leave-one-year-out procedure, analogous to bootstrapping, in which decarbonization trends, δ_i, and decarbonization rates, λ_i, are re-estimated after sequentially omitting one year from the sample. For each energy scenario—business-as-usual (linear), business-as-usual (exponential), transition (linear), and transition (exponential)—this results in five runs: one main run using all years 2020–2024 and four additional runs, each excluding a different year. This approach allows us to construct an uncertainty envelope around the main run, defined by the minimum and maximum values of the low-carbon share, l_i, across all runs. Supplementary Figure 13 illustrates the four energy scenarios, the leave-one-year-out runs, and the resulting uncertainty envelopes. Supplementary Table 6 reports the values of the main runs alongside the lower and upper bounds across all leave-one-year-out runs. Overall, the scenarios are remarkably stable under the leave-one-year-out analysis. The linear scenarios show only small differences between their minimum and maximum values. The exponential scenarios are also stable, though their upper bounds deviate more strongly from the main runs. This deviation arises when the year 2020 is excluded from the estimation, which increases the slope of some firms and leads to an uptick in the projected low-carbon share. This is intuitive, as the estimated low-carbon share in 2020 is higher than in the following years (2020–2024 observed period), so including 2020 in the estimation leads to more negative slopes. Despite this sensitivity, the close agreement of aggregate low-carbon shares across the different runs underscores the robustness and stability of our results.

Although we test the sensitivity of our results to the estimation period, we do not explicitly examine the assumption of extended average energy consumption, as introducing additional assumptions would substantially increase the complexity of interpreting the results. The scenario runs are intended primarily for illustration: they show that current firm-level decarbonization trends are insufficient to meaningfully change energy consumption, whereas meaningful reductions would be achievable if firms followed the frontrunners within their industries.

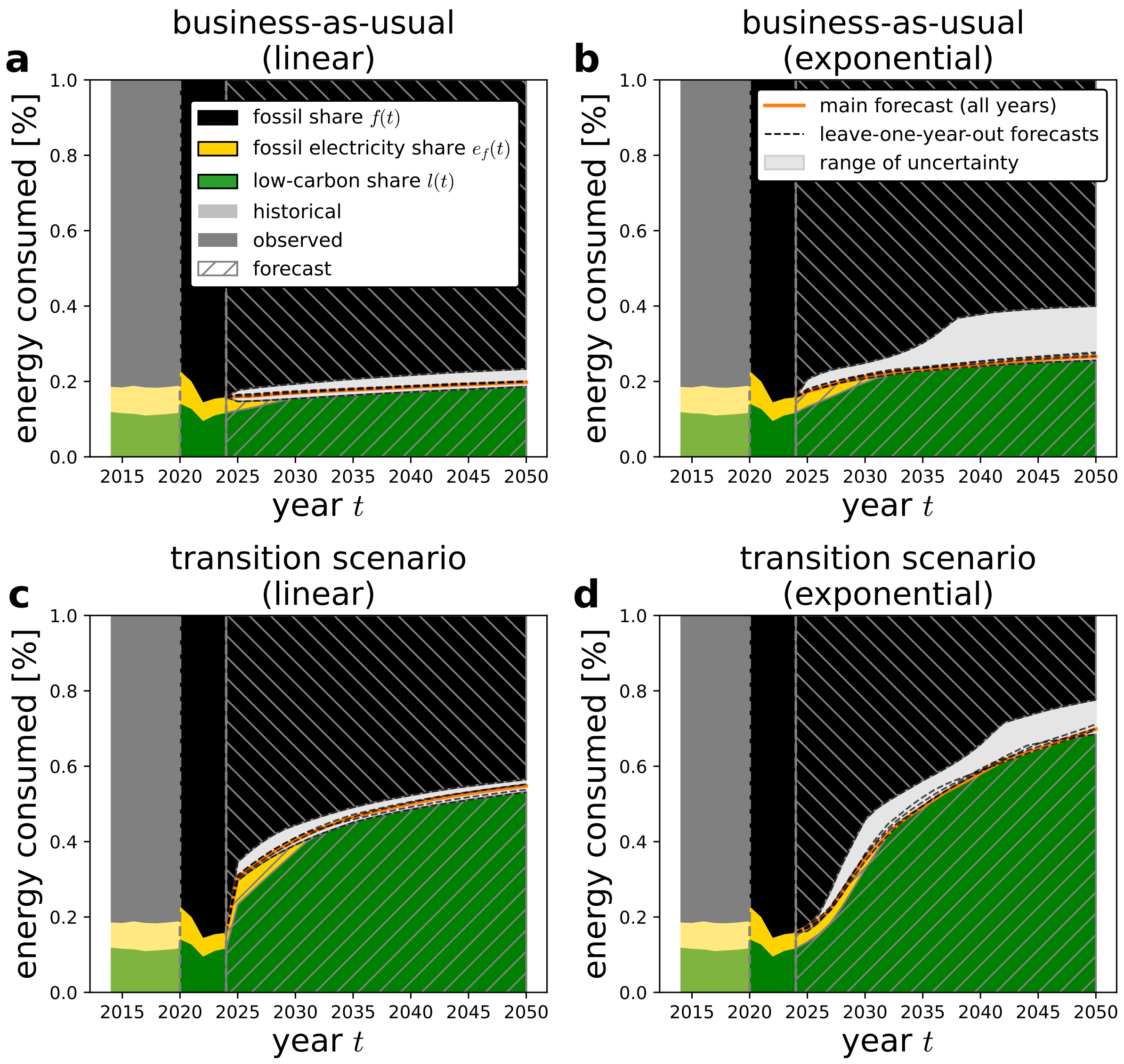

Supplementary Figure 13: Scenarios of aggregate energy consumption by fossil share, $f(t)$, fossil electricity share, $e_f(t)$, and low-carbon share, $l(t)$, based on observed firm-level trends. Grey shaded areas indicate the maximum uncertainty envelope across five runs per scenario: one baseline run using all available years (2020–2024) and four additional runs in which decarbonization trends or rates were re-estimated by leaving out one year at a time. Panels show business-as-usual scenarios (linear, a; exponential, b) and transition scenarios (linear, c; exponential, d). Exact values of the low-carbon and fossil shares for 2020, 2030, 2040, and 2050, together with the corresponding minimum and maximum values, are provided in Supplementary Table 6.

Supplementary Table 6: Aggregate low-carbon share, $l(t)$, and fossil share, $f(t)$, across the scenarios shown in Supplementary Figure 13, with associated uncertainty bounds.

	**business-as-usual (linear)**		**business-as-usual (exponential)**		**transition scenario (linear)**		**transition scenario (exponential)**	
year	$l(t)$	$f(t)$	$l(t)$	$f(t)$	$l(t)$	$f(t)$	$l(t)$	$f(t)$
2020	0.141	0.772	0.141	0.772	0.141	0.772	0.141	0.772
2030	$0.170^{+0.024}_{-0.015}$	$0.830^{+0.015}_{-0.024}$	$0.213^{+0.036}_{-0.005}$	$0.787^{+0.005}_{-0.036}$	$0.403^{+0.041}_{-0.012}$	$0.597^{+0.012}_{-0.041}$	$0.353^{+0.105}_{-0.004}$	$0.647^{+0.004}_{-0.105}$
2040	$0.185^{+0.032}_{-0.013}$	$0.815^{+0.013}_{-0.032}$	$0.245^{+0.133}_{-0.004}$	$0.755^{+0.004}_{-0.133}$	$0.500^{+0.022}_{-0.016}$	$0.500^{+0.016}_{-0.022}$	$0.582^{+0.076}_{-0.000}$	$0.418^{+0.000}_{-0.076}$
2050	$0.198^{+0.035}_{-0.012}$	$0.802^{+0.012}_{-0.035}$	$0.266^{+0.134}_{-0.010}$	$0.734^{+0.010}_{-0.134}$	$0.548^{+0.017}_{-0.017}$	$0.452^{+0.017}_{-0.017}$	$0.699^{+0.078}_{-0.014}$	$0.301^{+0.014}_{-0.078}$

Limitations in establishing causality between firm characteristics and transition behavior

A natural question arising from our analysis is whether specific firm characteristics *cause* firms to transition toward higher shares of low-carbon energy, or conversely, to remain locked-in to fossil-fuel–intensive portfolios. While our results identify patterns consistent with a lock-in mechanism, the empirical design does not allow us to determine the direction of causality.

To explore temporal precedence, we attempted to implement the heterogeneous panel Granger non-causality test proposed by Dumitrescu and Hurlin (2012) [9]. In principle, this method can identify whether changes in firm characteristics systematically precede changes in low-carbon shares across firms.

In practice, however, the test requires a considerably longer time-series dimension, typically $T \geq 10$, for stable estimation. Our dataset contains only five yearly observations per firm, leaving too few effective data points once lags are included. As a result, many firm-level regressions become numerically unstable, causing the test statistic Z_{bar} to diverge to unrealistically large values. In such cases, the p-values no longer provide meaningful evidence of causality but instead reflect the breakdown of the test under extremely short panels. Traditional time-series Granger tests suffer from the same limitation [10]. A future study with a longer time series could meaningfully revisit this analysis.

Given these limitations, our analysis should be viewed as descriptive: we document systematic differences between firms that increase their low-carbon energy share and those that do not, but we do not identify the mechanisms driving these differences. Future work could pursue causal identification by leveraging longer time-series data, natural or quasi-experiments (e.g., policy shocks, regulatory changes, eligibility thresholds), or event-study designs to examine dynamic responses to exogenous changes. Such approaches would provide a stronger foundation for causal claims about the determinants of firms' energy transition pathways.

Estimation of the effect of solar PV self-consumption

Our firm-level electricity consumption estimates are derived by aggregating monetary expenditures on electricity purchases from energy providers and converting them into kilowatt-hours. This measurement strategy does not capture behind-the-meter (BTM) electricity consumption, i.e. electricity generated and consumed on-site, which has become increasingly relevant due to the rapid expansion of solar photovoltaic (PV) capacity in Hungary during the observation period. PV installations on commercial premises commonly serve both self-consumption and grid feed-in.

To approximate the electricity we may be missing by ignoring BTM self-consumption, we draw on several complementary data sources.

The Hungarian Energy and Utilities Regulatory Authority (MEKH) publishes annual data of total gross PV electricity generation, denoted $G^{\text{MEKH}}(t)$, which include both grid-injected electricity and estimated production from smaller systems [11,12]. In contrast, the European Network of Transmission System Operators for Electricity (ENTSO-E) reports only electricity that actually enters the grid, denoted $G^{\text{grid}}(t)$, through its transparency platform [13]. Annual country-level aggregations of this dataset are provided by Energy Charts [14].

The Hungarian transmission system operator MAVIR provides a detailed breakdown of installed PV capacity [15], distinguishing: (i) household-scale systems (HMKE); (ii) utility-scale PV plants primarily feeding into the grid; and (iii) industrial-scale installations intended for self-consumption (SCTE). The same source reports annual PV capacity factors, ranging around 17%. Based on this classification, we assume that utility-scale PV systems feed all generated electricity into the grid, that SCTE installations generate exclusively for self-consumption by firms, and that a fraction of HMKE systems located on commercial buildings also produce electricity used by firms directly.

Let $C^{\text{SCTE}}(t)$ denote the installed annual SCTE capacity and $CF(t)$ the annual PV capacity factor. SCTE generation is then given by Supplementary Equation (1):

$$G^{\text{SCTE}}(t) = C^{\text{SCTE}}(t) \cdot CF(t) \cdot 8760. \tag{1}$$

Because electricity produced with SCTE systems is almost certainly consumed by industrial users, the resulting estimates of firms' low-carbon electricity shares may be distorted for industrial firms that have invested in this type of on-site generation.

A study by the market research company Forsense [16], drawing on MAVIR data, reports both total HMKE capacity, $C^{\text{HMKE}}(t)$, and residential HMKE capacity, $C^{\text{HMKE,res}}(t)$. From these data, we infer commercial HMKE

capacity as $C^{\text{HMKE,com}}(t) = C^{\text{HMKE}}(t) - C^{\text{HMKE,res}}(t)$. We adopt the conservative assumption that the entirety of commercial HMKE generation is self-consumed rather than fed into the grid. While plausible for larger industrial firms, this is unlikely to hold for smaller service-sector firms that typically both self-consume and feed into the grid. We use this restrictive assumption deliberately to derive an upper bound on the average measurement error arising from unobserved self-consumption. Commercial HMKE generation is thus defined as:

$$G^{\text{HMKE,com}}(t) = C^{\text{HMKE,com}}(t) \cdot CF(t) \cdot 8760. \tag{2}$$

Total commercial PV self-consumption is therefore given by Supplementary Equation (3):

$$G^{\text{SC,com}}(t) = G^{\text{SCTE}}(t) + G^{\text{HMKE,com}}(t), \tag{3}$$

and its share in total national PV generation is

$$s^{\text{PV,com}}(t) = \frac{G^{\text{SC,com}}(t)}{G^{\text{MEKH}}(t)}. \tag{4}$$

On average, commercial PV self-consumption accounts for approximately 27% of total annual PV generation over the observation period.

To relate commercially self-consumed PV electricity to total commercial electricity use, let $F^{\text{com}}(t)$ denote commercial electricity demand from the detailed national energy balance[5]. We compute this by subtracting residential consumption, $F^{\text{res}}(t)$ from total final electricity consumption, $F(t)$:

$$F^{\text{com}}(t) = F(t) - F^{\text{res}}(t). \tag{5}$$

The share of commercial total electricity consumption met through self-consumed PV is then

$$s^{\text{com}}(t) = \frac{G^{\text{SC,com}}(t)}{F^{\text{com}}(t)}, \tag{6}$$

which rises from approximately 2.3% in 2020 to 8.8% in 2024. Table 7 and Tab. 8 summarize the underlying data and calculated results.

Although this increase is substantial, its absolute magnitude remains modest relative to total commercial electricity demand, and the average impact is therefore likely to be limited. The impact is likely heterogeneous across sectors: service-sector firms may exhibit comparatively higher self-consumption shares relative to their overall electricity use, whereas large industrial firms typically consume much larger volumes of electricity, making the contribution of PV self-consumption relatively small. Consequently, our estimates for large industrial electricity consumers are unlikely to be materially affected. This may not hold for firms that have invested in SCTE solar PV systems; however, because we cannot identify these firms, this remains a limitation of our analysis. By contrast, the low-carbon electricity shares, $l_i(t)$ may be understated for smaller service-sector firms and should therefore be interpreted with caution.

Supplementary Table 7: Data and calculations to estimate commercial PV self-consumption for the years 2020-2024 [1]

year t	$C^{\text{util}}(t)$ (MW)	$C^{\text{SCTE}}(t)$ (MW)	$C^{\text{HMKE}}(t)$ (MW)	$C^{\text{HMKE,res}}(t)$ (MW)	$C^{\text{HMKE,com}}(t)$ (MW)	$G^{\text{MEKH}}(t)$ (GWh)	$G^{\text{grid}}(t)$ (GWh)	$G^{\text{SC}}(t)$ (GWh)
2020	1407.0	171.8	719.0	447.0	272.0	2459.0	1594.0	865.0
2021	1829.3	225.9	1125.0	757.0	368.0	3796.0	2399.0	1397.0
2022	2524.9	329.1	1492.0	1044.0	448.0	4732.0	3081.0	1651.0
2023	3301.7	407.8	2329.0	1638.0	691.0	6925.0	4399.0	2526.0
2024	4030.2	781.4	2540.0	1734.0	806.0	9200.0	5687.0	3513.0
source	MAVIR [15]	MAVIR [15]	Forsense [16]	Forsense [16]	derived	MEKH-1 [11]	Energy Charts [14] ENTSO-E [13]	derived

Supplementary Table 8: Data and calculations to estimate commercial PV self-consumption for the years 2020-2024 [2]

year t	$CF(t)$	$G^{\mathrm{SCTE}}(t)$ (GWh)	$G^{\mathrm{HMKE,com}}(t)$ (GWh)	$G^{\mathrm{SC,com}}(t)$ (GWh)	$s^{\mathrm{PV,com}}(t)$	$F^{\mathrm{total}}(t)$ (GWh)	$F^{\mathrm{res}}(t)$ (GWh)	$F^{\mathrm{com}}(t)$ (GWh)	$s^{\mathrm{com}}(t)$
2020	16.35%	246.7	389.6	636.3	25.88%	40046.0	12275.0	27771.0	2.29%
2021	16.87%	333.8	543.8	877.7	23.12%	41716.0	12475.0	29241.0	3.00%
2022	17.14%	672.7	672.7	1345.3	28.43%	41305.0	12348.0	28957.0	4.65%
2023	16.66%	1008.5	1008.5	2016.9	29.13%	40197.0	12532.0	27665.0	7.29%
2024	17.75%	1253.2	1253.2	2506.5	27.24%	40493.0	12089.0	28404.0	8.82%
source	MAVIR [15]	derived	derived	derived	derived	MEKH-2 [5]	MEKH-2 [5]	derived	derived

Influence of suppliers and customers on transitioning behaviour

We investigate firms' transition behavior, and how it is associated with the transition behavior of their suppliers and customers. Our analysis consists of two parts.

First, we compute the mean decarbonization trend of each firm's suppliers and customers, based on their individual trends $\overline{\delta_j}$, and correlate this mean with the firm's own decarbonization trend, δ_i. A positive correlation indicates that the decarbonization trends of firms and their partners are aligned (both increasing or both decreasing), whereas a negative correlation indicates that firms and their partners tend to move in opposite directions. We report Pearson and Spearman correlations in Supplementary Figure 14. The results show negative correlations between the mean trends of suppliers and focal firms across tiers, positive correlations between mean trends of customers and focal firms for tiers 1 and 2, and negative correlations for customers at tier 3. While the correlations are not large, the pattern suggests that firms' decarbonization trends are more closely associated with those of their direct customers than with those of their suppliers.

Second, we calculate the share of suppliers and customers that are transitioning for each firm, and correlate this share with the firm's own transition status. A positive correlation indicates that transitioning firms are associated with having a higher fraction of transitioning partners than non-transitioning firms, while a negative correlation indicates the opposite. We report Pearson and Spearman correlations in Figure 15. The results again show negative correlations between the share of transitioning suppliers and firms' own transition status, and positive correlations between the share of transitioning direct customers and firms' own transition status. This pattern suggests that firms' transition decisions are more closely associated with the behavior of their customers than with that of their suppliers, which is consistent with the interpretation that customer demand creates pressure to decarbonize.

These findings should be interpreted with caution. Because we only observe the transition status of firms in our sample, our measures of partner behavior are necessarily incomplete and exclude suppliers and customers outside the dataset. Moreover, the number of partners increases sharply with distance in the network, from typically around 100 in tier 1 to several thousand in tier 2 and more than 10,000 in tier 3. This scaling may influence the correlations and complicates comparisons across tiers. We therefore regard this analysis as exploratory, and it serves as motivation for future research on the relationship between firms' participation in the energy transition and the behavior of their suppliers and customers.

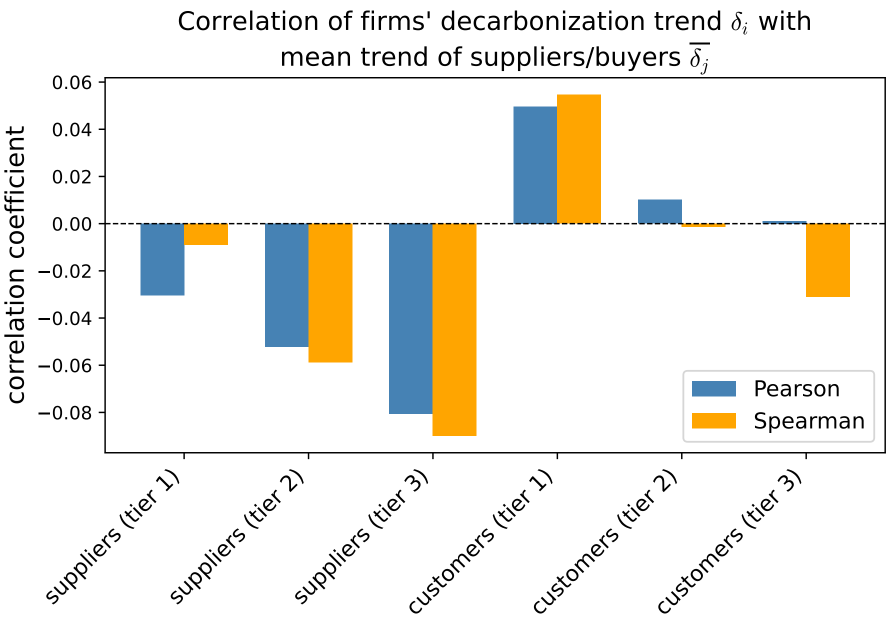

Supplementary Figure 14: Correlation between firms' decarbonization trends and the mean trends of their suppliers and customers. For each supply-chain tier (1–3), a firm's decarbonization trend, δ_i, is correlated with the mean trend of its suppliers and customers, $\overline{\delta_j}$. Pearson and Spearman correlation coefficients are reported separately for suppliers and customers at each tier.

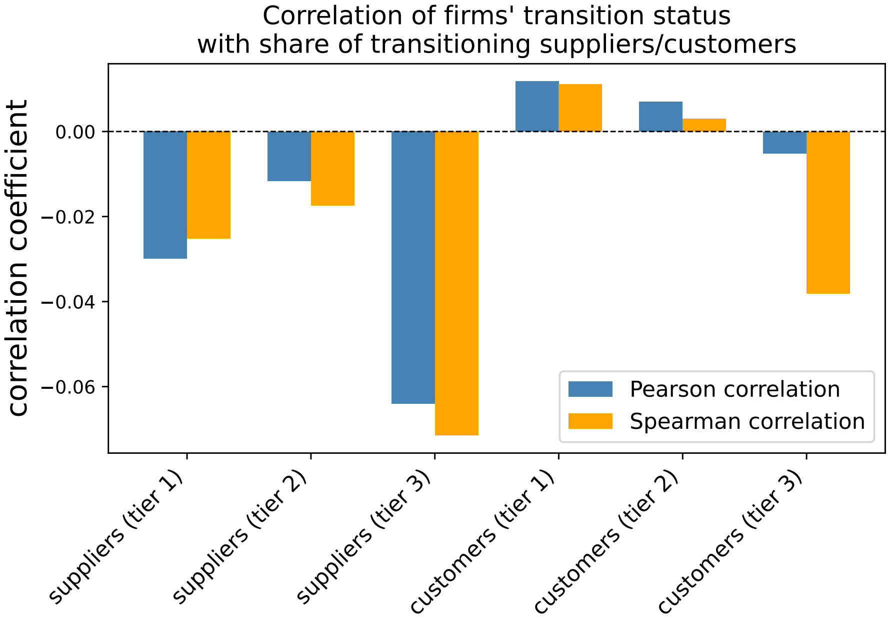

Supplementary Figure 15: Correlation between firms' transition status (transitioning/non-transitioning) and the share of their transitioning suppliers and customers. Pearson and Spearman correlation coefficients are reported separately for suppliers and customers across supply-chain tiers 1–3.

Supplementary References

[1] Ember. Electricity Data Explorer (2023). URL https://ember-energy.org/data/electricity-data-explorer/. Accessed August 18, 2025.

[2] Ministry for Innovation and Technology. National Clean Development Strategy (2020). URL https://unfccc.int/sites/default/files/resource/LTS_1_Hungary_2021_EN.pdf. Accessed December 18, 2024.

[3] Eurostat. Electricity prices for non-household consumers - bi-annual data (from 2007 onwards) (2024). URL https://ec.europa.eu/eurostat/databrowser/view/nrg_pc_205__custom_9257772/default/table?lang=en. Accessed August 18, 2025.

[4] Eurostat. Gas prices for non-household consumers - bi-annual data (from 2007 onwards) (2024). URL https://ec.europa.eu/eurostat/databrowser/view/nrg_pc_203__custom_12406662/default/table?lang=en. Accessed August 18, 2025.

[5] Hungarian Energy and Public Utility Regulatory Authority. National detailed energy balance - iea format - (annual) 2014-2024 (2025). URL https://www.mekh.hu/download/2/63/b1000/7_3_orszagos_eves_IEA_tipusu_reszletes_energiamerleg_2014_2024.xlsx. Accessed November 25, 2025.

[6] European Commission. Weekly oil bulletin price developments 2005 onwards (2024). URL https://energy.ec.europa.eu/document/download/906e60ca-8b6a-44e7-8589-652854d2fd3f_en?filename=Weekly_Oil_Bulletin_Prices_History_maticni_4web.xlsx. Accessed August 18, 2025.

[7] Huber, P. J. Robust estimation of a location parameter. *The Annals of Mathematical Statistics* **35**, 73–101 (1964). URL http://dx.doi.org/10.1214/aoms/1177703732.

[8] Hungarian Energy and Public Utility Regulatory Authority. 8.2 final energy use of industrial sector 2020-2023 (2025). URL https://www.mekh.hu/download/f/a1/91000/8_2_Ipar_vegso_felhasznalas_reszletes_eves_2020_2023.xlsx. Accessed September 14, 2025.

[9] Dumitrescu, E.-I. & Hurlin, C. Testing for granger non-causality in heterogeneous panels. *Economic Modelling* **29**, 1450–1460 (2012). URL http://dx.doi.org/10.1016/j.econmod.2012.02.014.

[10] Granger, C. W. J. Investigating causal relations by econometric models and cross-spectral methods. *Econometrica* **37**, 424 (1969). URL http://dx.doi.org/10.2307/1912791.

[11] Hungarian Energy and Public Utility Regulatory Authority. 4.2 annual data for gross electricity generation 2014-2024 (2025). URL https://www.mekh.hu/download/d/43/b1000/4_2_brutto_villamos_energia_termeles_eves_2014_2024.xlsx. Accessed November 25, 2025.

[12] Hungarian Energy and Public Utility Regulatory Authority. Methodology: 4.2 annual data for gross electricity generation (2025). URL https://www.mekh.hu/download/7/bf/01000/4_2_brutto_villamos_energia_termeles.pdf. Accessed December 2, 2025.

[13] ENTSO-E. Entso-e transparency platform (2025). URL https://transparency.entsoe.eu/. Accessed November 25, 2025.

[14] Fraunhofer Institute for Solar Energy Systems ISE. Public net electricity generation in hungary in november 2025 (2025). URL https://energy-charts.info/charts/energy/chart.htm?l=en&c=HU. Accessed November 25, 2025.

[15] MAVIR Zrt. Pv statisztika. https://mavir.hu/documents/10258/291845564/PV+STATISZTIKA_20250101-ig_v1_HU.pdf (2025). Accessed November 25, 2025.

[16] forsense. Megújuló energiaforrásokkal kapcsolatos trendek és vélemények magyarországon (2024). URL https://kekbolygoalapitvany.hu/wp-content/uploads/2025/02/202412_Megujulo-energiaforrasok_KBA_2024_.pdf. Accessed November 25, 2025.
