# Supplementary material for: Using firm-level supply chain networks to measure the speed of the energy transition
Source: Nat Commun. 2026 Feb 9;17:2529. doi: 10.1038/s41467-026-69358-4 (PMC12996292; doi:10.1038/s41467-026-69358-4)
Supplement: Supplementary file 2 — Reporting Summary [file 41467_2026_69358_MOESM2_ESM.pdf]

nature portfolio

Corresponding author(s): Stefan Thurner
Last updated by author(s): Jan 14, 2026

# Reporting Summary

Nature Portfolio wishes to improve the reproducibility of the work that we publish. This form provides structure for consistency and transparency in reporting. For further information on Nature Portfolio policies, see our Editorial Policies and the Editorial Policy Checklist.

## Statistics

For all statistical analyses, confirm that the following items are present in the figure legend, table legend, main text, or Methods section.

| n/a | Confirmed | |
|---|---|---|
| ☐ | ☒ | The exact sample size (*n*) for each experimental group/condition, given as a discrete number and unit of measurement |
| ☐ | ☒ | A statement on whether measurements were taken from distinct samples or whether the same sample was measured repeatedly |
| ☐ | ☒ | The statistical test(s) used AND whether they are one- or two-sided<br>*Only common tests should be described solely by name; describe more complex techniques in the Methods section.* |
| ☐ | ☒ | A description of all covariates tested |
| ☐ | ☒ | A description of any assumptions or corrections, such as tests of normality and adjustment for multiple comparisons |
| ☐ | ☒ | A full description of the statistical parameters including central tendency (e.g. means) or other basic estimates (e.g. regression coefficient) AND variation (e.g. standard deviation) or associated estimates of uncertainty (e.g. confidence intervals) |
| ☒ | ☐ | For null hypothesis testing, the test statistic (e.g. *F*, *t*, *r*) with confidence intervals, effect sizes, degrees of freedom and *P* value noted<br>*Give P values as exact values whenever suitable.* |
| ☒ | ☐ | For Bayesian analysis, information on the choice of priors and Markov chain Monte Carlo settings |
| ☒ | ☐ | For hierarchical and complex designs, identification of the appropriate level for tests and full reporting of outcomes |
| ☐ | ☒ | Estimates of effect sizes (e.g. Cohen's *d*, Pearson's *r*), indicating how they were calculated |

*Our web collection on statistics for biologists contains articles on many of the points above.*

## Software and code

Policy information about availability of computer code

| Data collection | R environment:<br>R (v4.2.2); RStudio (v2023.06.1);<br>R packages: data.table (v1.14.8), igraph (v1.5.0), Matrix (v1.5.1).<br><br>Python environment:<br>Python (v3.12.3); Visual Studio Code (v1.108.0); Jupyter Server (v2.14.1);<br>Python packages: numpy (v1.26.4), pandas (v2.2.2). |
|---|---|

| Data analysis | R environment:<br>R (v4.2.2); RStudio (v2023.06.1);<br>R packages: data.table (v1.14.8), igraph (v1.5.0), Matrix (v1.5.1).<br><br>Python environment:<br>Python (v3.12.3); Jupyter Server (v2.14.1); Visual Studio Code (v1.108.0);<br>Python packages: numpy (v1.26.4), pandas (v2.2.2), matplotlib (v3.9.0), plotly (v5.22.0), scikit-learn (v1.5.2), statsmodels (v0.14.4), seaborn (v0.13.2).<br><br>Custom code developed for this study: https://github.com/jo-stangl/using_firm-level_supply_chain_networks_to_measure_the_speed_of_the_energy_transition |
|---|---|

For manuscripts utilizing custom algorithms or software that are central to the research but not yet described in published literature, software must be made available to editors and reviewers. We strongly encourage code deposition in a community repository (e.g. GitHub). See the Nature Portfolio guidelines for submitting code & software for further information.

## Data

Policy information about availability of data

All manuscripts must include a data availability statement. This statement should provide the following information, where applicable:

- Accession codes, unique identifiers, or web links for publicly available datasets
- A description of any restrictions on data availability
- For clinical datasets or third party data, please ensure that the statement adheres to our policy

The raw data on financial transactions between Hungarian value-added tax paying firms are protected and are not available due to data privacy laws. Requests for collaborations to work with these data can be addressed to \url{olahzs@mnb.hu}.

## Research involving human participants, their data, or biological material

Policy information about studies with human participants or human data. See also policy information about sex, gender (identity/presentation), and sexual orientation and race, ethnicity and racism.

| Reporting on sex and gender | - |
|---|---|
| Reporting on race, ethnicity, or other socially relevant groupings | - |
| Population characteristics | - |
| Recruitment | - |
| Ethics oversight | - |

Note that full information on the approval of the study protocol must also be provided in the manuscript.

# Field-specific reporting

Please select the one below that is the best fit for your research. If you are not sure, read the appropriate sections before making your selection.

☐ Life sciences ☒ Behavioural & social sciences ☐ Ecological, evolutionary & environmental sciences

For a reference copy of the document with all sections, see nature.com/documents/nr-reporting-summary-flat.pdf

# Behavioural & social sciences study design

All studies must disclose on these points even when the disclosure is negative.

| Study description | Quantitative study |
|---|---|
| Research sample | The research sample consists of 25,231 value-added tax–paying firms in Hungary. The sample was constructed from administrative transaction records and includes firms meeting predefined criteria on the continuity of time-series observations for revenues and transactions attributable to energy providers (see Methods). Firms operating in energy-supply sectors and financial institutions were excluded, as the analysis focuses on final energy use. |
| Sampling strategy | The study uses a non-probability, inclusion-criteria–based sampling strategy applied to administrative transaction records of Hungarian value-added tax–paying firms. Firms were included if they satisfied predefined requirements on the continuity of time- |

| | |
|---|---|
| | series observations for revenues and transactions attributable to energy providers (see Methods). Firms operating in energy-supply sectors and financial institutions were excluded, as the analysis focuses on final energy use. |
| Data collection | The study is based on administrative transaction records of Hungarian value-added tax–paying firms. Data were collected electronically via centralized governmental tax reporting systems. No primary data were collected directly from human participants. |
| Timing | November 2023 until August 2025 |
| Data exclusions | One firm was excluded from the sample due to likely misreporting. Specifically, the aggregated transaction values from entities labeled as gas providers exhibited an abrupt increase of approximately three orders of magnitude within a single year, while values in all other years were of comparable magnitude. This pattern was inconsistent with plausible economic activity and was therefore treated as a data error. |
| Non-participation | Not applicable. This study did not involve recruitment of human participants; all analyses were conducted on existing administrative records. |
| Randomization | Not applicable. This study did not involve experimental assignment or manipulation; all analyses were conducted on existing administrative records. |

# Reporting for specific materials, systems and methods

We require information from authors about some types of materials, experimental systems and methods used in many studies. Here, indicate whether each material, system or method listed is relevant to your study. If you are not sure if a list item applies to your research, read the appropriate section before selecting a response.

## Materials & experimental systems

| n/a | Involved in the study | |
|---|---|---|
| ☒ | ☐ | Antibodies |
| ☒ | ☐ | Eukaryotic cell lines |
| ☒ | ☐ | Palaeontology and archaeology |
| ☒ | ☐ | Animals and other organisms |
| ☒ | ☐ | Clinical data |
| ☒ | ☐ | Dual use research of concern |
| ☒ | ☐ | Plants |

## Methods

| n/a | Involved in the study | |
|---|---|---|
| ☒ | ☐ | ChIP-seq |
| ☒ | ☐ | Flow cytometry |
| ☒ | ☐ | MRI-based neuroimaging |

## Plants

| | |
|---|---|
| Seed stocks | -- |
| Novel plant genotypes | -- |
| Authentication | - |
